# The Prevalence and Factors Associated with the Prescription of Opioids for Head/Neck Pain after Elective Craniotomy for Tumor Resection/Vascular Repair: A Retrospective Cohort Study

**DOI:** 10.3390/medicina59010028

**Published:** 2022-12-23

**Authors:** Wei-Yun Wang, Varadaraya Satyanarayan Shenoy, Christine T. Fong, Andrew M. Walters, Laligam Sekhar, Michele Curatolo, Monica S. Vavilala, Abhijit V. Lele

**Affiliations:** 1 Department of Anesthesiology and Pain Medicine, University of Washington, Seattle, WA 98104, USA; 2 Department of Neurological Surgery, University of Washington, Seattle, WA 98104, USA

**Keywords:** analgesia, craniotomy, opioids, pain, length of stay

## Abstract

*Background and objective:* There is no report of the rate of opioid prescription at the time of hospital discharge, which may be associated with various patient and procedure-related factors. This study examined the prevalence and factors associated with prescribing opioids for head/neck pain after elective craniotomy for tumor resection/vascular repair. *Methods*: We performed a retrospective cohort study on adults undergoing elective craniotomy for tumor resection/vascular repair at a large quaternary-care hospital. We used univariable and multivariable analysis to examine the prevalence and factors (pre-operative, intraoperative, and postoperative) associated with prescribing opioids at the time of hospital discharge. We also examined the factors associated with discharge oral morphine equivalent use. *Results*: The study sample comprised 273 patients with a median age of 54 years [IQR 41,65], 173 females (63%), 174 (63.7%) tumor resections, and 99 (36.2%) vascular repairs. The majority (*n* = 264, 96.7%) received opioids postoperatively. The opiate prescription rates were 72% (*n* = 196/273) at hospital discharge, 23% (19/83) at neurosurgical clinical visits within 30 days of the procedure, and 2.4% (2/83) after 30 days from the procedure. The median oral morphine equivalent (OME) at discharge use was 300 [IQR 175,600]. Patients were discharged with a median supply of 5 days [IQR 3,7]. On multivariable analysis, opioid prescription at hospital discharge was associated with pre-existent chronic pain (adjusted odds ratio, aOR 1.87 [1.06,3.29], *p* = 0.03) and time from surgery to hospital discharge (compared to patients discharged within days 1–4 postoperatively, patients discharged between days 5–12 (aOR 0.3, 95% CI [0.15; 0.59], *p* = 0.0005), discharged at 12 days and later (aOR 0.17, 95% CI [0.07; 0.39], *p* < 0.001)). There was a linear relationship between the first 24 h OME (*p* < 0.001), daily OME (*p* < 0.001), hospital OME (*p* < 0.001), and discharge OME. *Conclusions*: This single-center study finds that at the time of hospital discharge, opioids are prescribed for head/neck pain in as many as seven out of ten patients after elective craniotomy. A history of chronic pain and time from surgery to discharge may be associated with opiate prescriptions. Discharge OME may be associated with first 24-h, daily OME, and hospital OME use. Findings need further evaluation in a large multicenter sample. The findings are important to consider as there is growing interest in an early discharge after elective craniotomy.

## 1. Introduction

According to the Centers for Disease Control, it is estimated that national opioid prescription in the US has tripled since 1999, and the economic cost of prescription opioid misuse exceeds 78 billion dollars annually [1]. In 2015, 30,000 people died of an opioid drug overdose in the US [2] while 67,400 died in 2018 [3]. Most opioid abuse originates from the misuse of prescription medication used to treat chronic pain [4,5]. Alarmingly, persistent postoperative pain occurs in as many as 10–50% of surgical patients [4], which puts these patients at high risk of developing opioid medication-related adverse events with additional healthcare costs [6].

Pain from craniotomy surgeries is commonly managed intraoperatively with fentanyl, remifentanil, or sufentanil infusions, intravenous fentanyl, morphine hydromorphone, and acetaminophen, as well as with the use of scalp blocks and infiltration of a local anesthetic at the craniotomy incision site. Even though it has been historically reported that post-craniotomy pain may be less severe compared to that after non-craniotomy procedures [7], several studies have demonstrated that acute post-craniotomy headaches can be severe and occur in as many as one-third to half of postoperative patients [8,9,10,11]. Some reasons for persistent post-craniotomy pain may include dural irritation, pericranial muscle retraction, surgical trauma, decreased cerebrospinal fluid pressure, persistent tension headache, and neck muscle spasm related to the patient’s position during the surgical intervention, and aseptic meningitis [5,12]. The location of surgical intervention may also be related to increased postoperative pain, with infratentorial surgeries having higher pain scores than supratentorial surgeries [13]. Despite using non-opioid analgesic techniques such as local anesthetic skin infiltration and/or scalp blocks and non-opioid medications, opioids remain the mainstay therapy for post-craniotomy headaches. While prior research has focused on intraoperative anesthetic management and pain experiences during their hospital stay [14,15,16,17], there is no report of the rate of opioid prescription at the time of hospital discharge, which may be associated with various patient and procedure-related factors.

To bridge this knowledge gap, we undertook a study to examine the prevalence and factors associated with opioid prescriptions in patients at the time of hospital discharge. We attempted to examine the continued prescription of opioids at the neurosurgical clinic follow-up. We studied this in a cohort of patients undergoing craniotomy for tumor resection/vascular repair. The specific aims of this study were (1) to examine the prevalence of opioid prescription at the time of hospital discharge and neurosurgical clinical follow-up within 30 days of the neurosurgical procedure and after 30 days of the neurosurgical procedure and (2) to examine the association between perioperative factors associated with the rate of opioid prescriptions. Knowledge gained from this study may increase awareness of opiate prescriptions amongst healthcare providers routinely caring for patients undergoing craniotomy, which in turn may inform responsible and appropriate discharge opioid prescription practices.

## 2. Materials and Methods

### 2.1. Study Design

This is a single-center, retrospective observational study conducted at Harborview Medical Center in Seattle, Washington, a comprehensive stroke, and Level I trauma center.

### 2.2. Patient Population

We included patients 18 years of age and older who underwent elective craniotomy by a single neurosurgeon (Author LS) at Harborview Medical Center between January 2015 and December 2019. We included craniotomy for tumor resections and vascular repair (microsurgical aneurysm repair, resection of arteriovenous malformation, and intracranial/extracranial bypass). Exclusion criteria included non-intracranial surgeries, emergency surgeries, more than one neurosurgical procedure during the same surgical encounter, expired patients, and patients with pain not located in the head/neck region.

### 2.3. Institutional Practice

All post-craniotomy patients were admitted to an intensive care unit, regardless of extubation status. None of the patients received scalp nerve blocks perioperatively. The pain was assessed by bedside nursing staff with the Critical-care Pain Observation Tool (CPOT) in intubated patients and by a numerical scale (range 0–10) in communicative patients.

### 2.4. Data Collection

Patient information and specific data are collected from the electronic health record (EHR) and refined via a departmental database, Perioperative & Pain Initiatives in Quality Safety Outcome (PPIQSO).

We categorized the variables of interest into three groups:

(a) Preoperative: age, sex, race category (White/non-White), ethnicity (Hispanic or Latino/Non-Hispanic/Non-Latino), Language category (English-speaking/Non-English speaking), insurance (Commercial/Non-commercial), history of chronic pain, pre-admission use of opiates, and oral morphine equivalent (OME), history of any depression/anxiety disorder, and a history of substance abuse.

(b) Intraoperative: type of procedure (tumor/vascular), location (supratentorial/infratentorial), surgical duration, use of remifentanil infusion, fentanyl infusion, lidocaine infusion, methadone, hydromorphone, ketamine infusion, and non-opioid analgesic such as intravenous acetaminophen.

(c) Post-operative: first 24 h post-op OME, first 24 h post-op maximum pain scores, maximum pain score during the hospital stays, daily OME from postoperative day one to postoperative day 30, total hospital OME, hospital OME/per day of stay in the hospital, and hospital length of stay.

(d) Follow-up: timing of visits in days since the original neurosurgical procedure and opiate prescriptions.

### 2.5. Outcome Measures

Opiates at discharge, OME at discharge, and Opiates prescribed at follow-up (within 30 days and 30 days after the original neurosurgical procedure). Total discharge OME and discharge OME per day is inferred from the number and dose of tablets and the administration frequency in the prescription. The OME conversion was as follows: oral morphine: conversion factor 1, oral controlled release Morphine: 1, oral codeine: 0.15, oral hydromorphone: 4, oral hydrocodone: 1, oral oxycodone: 1.5, oral oxymorphone: 3, oral meperidine: 0.1, oral levorphanol: 15, oral tramadol: 0.25, oral tapentadol: 0.3, transdermal fentanyl: (mcg/h): 2.4, oral methadone (1–20 mg/day): 4, oral methadone (21–40 mg/day): 8, oral methadone (41–60 mg/day): 10, oral methadone (>61–80 mg/day): 12, sublingual buprenorphine: 30.

### 2.6. Data Analysis and Statistics

The descriptive analysis described the study sample characteristics. After normality testing using the Shapiro–Wilk test, continuous variables such as age, body mass index (BMI), 24 h OME, hospital OME, OME at discharge, and hospital length of stay were expressed as median [interquartile range, IQR]. Categorical variables were expressed as counts and percentages.

Univariable analysis to examine associations with opiate prescription at hospital discharge was performed using the following parameters: age, sex, ASA physical class, insurance type, type of case (tumor/vascular), location (supratentorial/infratentorial), race category (non-white/white), ethnicity category (declined/unknown/Hispanic or Latino/not Hispanic or Latino, language category (English/non-English), history of chronic pain, history of a depression/anxiety disorder, history of substance abuse, preoperative opioid use, preoperative OME, length of surgical procedure, intraoperative ketamine infusion, hospital OME, hospital LOS in days: 1–4 days/5–12 days/>12 days. Multivariable analysis was performed using the following factors: intraoperative ketamine infusion, preoperative opioid use, and hospital LOS (days): 1–4 days/5–12 days/>12 days. Hosmer-Lemeshow goodness-of-fit test was performed on the multivariable model. Comparisons between patients receiving opioids at the time of hospital discharge and those that did not were performed using the Wilcoxon test for continuous variables and the Chi-square test for categorical variables. Odds ratio (OR) and 95% confidence intervals (CI) were calculated to examine the odds of receiving opioids at the time of discharge. A linear regression model examined the correlation between the first-24 h OME, daily OME, hospital LOS and discharged OME use. We also examined the association between discharge OME age, sex, preoperative opioid use, history of psychiatric disorder, history of chronic pain, and the use of ketamine infusion intraoperatively. A Bonferroni-corrected *p*-value of <0.05 indicates statistical significance. STATA 15 [18]/RStudio version 1.554 [19] was used for statistical analysis.

## 3. Results

### 3.1. Study Sample Characteristics

Table 1 provides details of the study sample characteristics. The study sample had 273 patients. The median age was 54 years [IQR 41, 63]. Overall, 161 (59%) patients had a chronic pain diagnosis; 123 (45%) patients had a pre-operative diagnosis of depression or anxiety; 66 (22%) patients had a pre-operative diagnosis of substance use disorder; 23 (8.4%) patients had a prescription for opioid medication in the 30 days before surgery. The majority (98.2%, *n* = 268) received a total intravenous anesthetic. Patients received remifentanil infusion (98.2%, *n* = 268), fentanyl infusion (0.7%, *n* = 2), ketamine infusion (31.7%, *n* = 87), intravenous acetaminophen (66.7%, *n* = 182), hydromorphone (21.2%, *n* = 58). No patient received morphine/methadone intraoperatively. The median length of surgery was 331.6 min [IQR 257,399]. Patients spent a median of 2 days [IQR 1,3] in the ICU and a median of 6 days [IQR 4,12] in the hospital. Eighty-three patients followed up in the neurosurgical clinic after the original neurosurgical procedure. The median time to follow-up time after the procedure at the neurosurgery clinic was 16 days [IQR 13–54], with the longest follow-up at 206 days. The median pain scores were 8[IQR 6,10].

### 3.2. Discharge and Follow-Up Opioid Prescriptions

One hundred ninety-six (72%) were prescribed opioids at hospital discharge. Opioids commonly prescribed included oxycodone (*n* = 170/196, 87%) and hydromorphone (*n* = 20/196, 10%). Others had tramadol (*n* = 3), methadone, morphine, and fentanyl (*n* = 1). The average total discharge OME was 45 [IQR 30,60]. Patients were discharged with a median supply of 5 days [IQR 3,7]. We found a linear relationship between days of discharge opioid supplied and 24 h postoperative OME use (Days of opioid supplied=4.06+0.01∗ 24−hour OME, R square=0.07, p<0.001), daily hospital OME use (Days of opioid supplied=3.49+0.04∗daily OME+R square=0.21, p<0.001), and total hospital OME (Days of opioid supplied=4.557+0.001∗Hospital OME, R square=0.044, p=0.003).

Amongst the 83 patients that followed up in the neurosurgical clinic at our institution, the opioid prescription rate during the first visit before 30 days of the neurosurgical procedure was 23% (*n* = 19), while the rate of opioid prescription after 30 days from the neurosurgical procedure was 2.4% (*n* = 2).

### 3.3. Factors Associated with Opioid Prescription at Hospital Discharge

Table 2 compares the groups of patients discharged on opioids to those discharged without an opioid prescription and provides univariable and multivariable analysis results. Opioid prescription at hospital discharge was associated with a history of chronic pain (adjusted odds ratio, aOR 1.87 [1.06,3.29], *p* = 0.03) and time from surgery to hospital discharge. Compared to patients discharged within days 1–4 postoperatively, patients discharged between days 5–12 (aOR 0.3, 95% CI [0.15; 0.59], *p* < 0.001), and at 12 days and later (aOR 0.17, 95% CI [0.07; 0.39], *p* <0.001) were less likely to be prescribed opioids at the time of discharge. The Homer-Lemeshow test (*p* = 0.27) indicated that the model adequately described the data. We found no association between discharge opioid prescription rate and total hospital OME (OR 1.00 [1.00; 1.00], *p* = 0.05).

### 3.4. Factors Associated with Discharge OME

**Figure 1:**
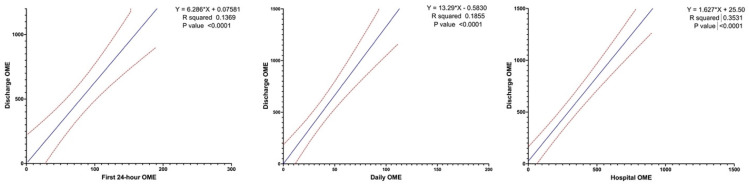
Correlation between first 24-h, Daily, and Total Hospital Oral Morphine Equivalent Use (OME) and Discharge Oral Morphine Equivalent Use.

Amongst those discharged on opioid medications (*n* = 196), the average total discharge OME was 300 [IQR 175,600]. The median first 24 h OME use was 58 [IQR 28,103]. The median daily OME was 23 [IQR 9,43]. The median total hospital OME was 178 [IQR 68,358]. There was a statistically significant correlation between discharge OME and (a) first 24 h OME (Discharge OME=0.07581+6.286∗24 h OME, R square=0.1369, p<0.001), (b) daily OME (Total discharge OME=13.29∗daily OME−0.5830, R square=0.1855, p<0.001), and (c) total hospital OME (Discharge OME=1.627∗Hospital OME+25.50, R square=0.3531, p<0.001). Figure 1 displays the correlation between first 24 h OME, daily OME, total hospital OME, and discharge OME.

There were no statistically significant differences in the discharge OME and history of chronic pain (509 ± 6 vs. 408 ± 113, *p* = 0.47) and discharge time after surgery (1–4 days: 432 ± 330 vs. 5–12 days: 422 ± 431 vs. >12 days: 759 ± 2372, *p* = 0.21)

## 4. Discussion

We examined the opioid burden and the prescription of opiates for head and neck pain at the time of hospital discharge after elective craniotomy for tumor resection/vascular procedures. The main findings of the study are described and discussed below.

Our study finds that seven out of ten patients are prescribed opioids at the time of their discharge from the hospital. Those with a history of pre-operative chronic pain were more likely to be prescribed opioids at discharge. In contrast, those with more extended hospital LOS were less likely to be prescribed opioids at discharge. Practitioners were more likely to prescribe patients with pre-existent chronic pain opioid pain medications on discharge since the presence and severity of pre-existing pain may correlate with the intensity of acute postoperative pain [20]. Given the lack of association between hospital OME and whether the patient is discharged with an opioid, there is a high likelihood that this trend may stem from medical practitioner perception rather than patient condition as a primary driver of opioid prescribing practices. For example, Brandal et al. looked at colorectal surgery. They found that 70% of opioid-naive patients with below-average opioid use during their hospital stay and low pain scores on discharge still received an opioid prescription upon discharge [21]. Since there is no institutional protocol with respect to the prescribing OME, the dose prescribed may very well be influenced by the subjective opinion of the treating physician, a finding observed in the neurocritical care setting [22]. Our study finds that opioid use is common in the postoperative period [23]. While in a small sample study, opioid-free craniotomy [24] was found to be non-inferior to traditional pain control concerning pain scores in the postoperative period, opioid-free post-craniotomy pain management remains elusive and warrants further examination. Only a minority of patients remained “opioid-free” during their hospital stay, given the “severe” pain cited by a substantial proportion of patients during their post-craniotomy period. Some patients may remain opioid-free, and others require a high OME rate. Expectations of pain and pain-control/pain-free state, variability in surgical techniques, location of the surgical site, and institutional pain regimens for an intraoperative and postoperative period may affect the frequency and severity of OME use.

With regard to the amount of opioid pain medications with which patients were discharged among those who were prescribed any after their craniotomy procedure, we found a statistically significant linear relationship between discharge OME and the first 24 h OME, daily OME, and total hospital OME. This may explain one of the reasons providers may base the amount of discharge OME to be prescribed. Though rich in risk factors for postoperative pain and the development of chronic pain, literature is scarce on factors associated with the amount of opioid medications patients are discharged with.

One observation that was not expected is the association between hospital length of stay and the rate of opioid prescription. The highest risk for opiate prescription in patients discharged within the first four days of their craniotomy, and its decrease with longer hospital length of stay suggest an association between pain scores and hospital OME, both of which decrease over time as the patient is observed after craniotomy. This would also be congruent with current studies, which showed that severe pain is commonly experienced within the first 48 h after surgery [13], presumably leading to an increased need for opioid pain medication, whether inpatient or upon discharge. In addition, when patients are discharged early, providers may fear that they will continue to have pain after leaving the hospital and are more likely to prescribe opioids on discharge. The literature has no comparable evidence regarding the association between LOS and opioid prescription in craniotomy cases. A study on abdominal transplant surgery found that LOS was protective against opioid over-prescription, likely because the initial postoperative opioid-requiring phase and/or acute complications have subsided [25]. On the other hand, other studies found that the longer the patients stay in the hospital after spine surgery [26], or wrist and ankle surgery [27], the more likely they are to be prescribed opioids at discharge.

Findings from our study suggest that hospital discharge opioid prescription was not associated with age, sex, prior opioid use, surgical location and approach, and history of depression and/or anxiety, which were thought to predispose patients to postoperative pain and opioid use [5,13,20,28]. Specifically, pre-existing opioid use, female gender, and young age were shown to be associated with generally more postoperative opioid use [5,20]. However, a study with a small cohort found that the male (rather than female) gender can be associated with increased postoperative pain [28]. In craniotomy surgeries, infratentorial approaches are associated with more postoperative pain than supratentorial approaches [13]. The parameter of ‘pre-existing opioid use’ in some of those studies referred to chronic pain, which would make our finding consistent with existing literature. Conversely, suppose a patient was on a short preoperative course of opioid pain medication for pain related to their intracranial pathology. In that case, the surgery should theoretically relieve the patient of the cause of their pain and therefore make prolonged postoperative pain unlikely.

At this time, opioid-free craniotomy care remains elusive [21,29,30], yet aspirational. To achieve an opioid-free state, there needs to be a shared mental model of understanding between the patient, the anesthesiologist, the neurosurgeon, and all postoperative clinicians. While alarmed by the opioid epidemic, we would be remiss to look at this as a potential pathway to craniotomy care. Recent studies published regarding the use of non-opiates in the routine management of craniotomy care indicate that while more data is awaited, perioperative clinicians have been interested in this.

This study puts into perspective the use of opioids after elective craniotomy and highlights possible reasons for prescribing opioids at the time of discharge. The findings of hospital LOS being an important factor raises concerns since there is growing interest in early discharge from the hospital after craniotomy [31,32]. Suppose patients are discharged earlier than historical controls. In that case, this may lead to an increased rate of opioid prescription due to the higher severity of pain and OME use in the first few days after craniotomy. Practitioners who are routinely involved in the care of these patients must balance the risks and benefits of prescribing opioids and the total amount of opiates prescribed for the post-discharge period. The findings of this study may be helpful in educating practitioners as well as patients.

### Limitations

Limitations are typical of a single-center retrospective study in that the data used for this study was not collected for research purposes, the sample size and power analysis were not performed a priori, and the follow-up data is missing more than half of the original sample size. If a patient received opioid-based patient-controlled analgesia during their postoperative period, there were institutional limitations regarding the accuracy of the recorded dosage. In addition, we could not assess if patients self-administered the opioids they were prescribed at discharge. We did not study the impact of patient positioning or tumor grading [33] on the rate of opioid prescription, nor can we say if prescription patterns for opioids may be same in a US-based vs. non-US-based sample where the prescription standards may vary. The study’s strengths are that we created a homogenous group of patients operated on by only one neurosurgeon, thus trying to reduce variability in surgical exposure and its potential effect on postoperative pain. We also restricted our study to head and neck pain, which, while excluding 20% from our original cohort, the final study sample was reflective of pain related to the surgical procedure. We also excluded those patients who had a non-neurosurgical procedure not to confound the potential reasons for using opiates for non-head and neck pain.

Being a retrospective study, findings are hypothesis-generating, and conclusions cannot be generalized to other institutions; however, the results from our research warrant a multicenter investigation in a larger cohort of craniotomy patients.

## 5. Conclusions

Opioids are commonly prescribed intra- and postoperatively after craniotomy. Most patients, as many as 7 in 10, may be prescribed opioids at the time of hospital discharge, though only 4 out of 100 patients are prescribed opioids 30 days after craniotomy.

## Figures and Tables

**Table 1:** Study Sample Characteristics.

	Overall (*n* = 273)
Age in years, Median [IQR]	54.0 [41; 63]
Female Sex	173 (63.4%)
ASA Class, Median [IQR]	3 [2; 3]
Commercial Insurance	144 (52.7%)
Type of Case	
Tumor resection	174 (63.7%)
Posterior-fossa tumor	77(44.3%)
Anterior/middle fossa skull base tumor	75(43.1%)
Convexity/parasagittal tumor	22(12.6%)
Vascular repair	99 (36.3%)
Cerebral aneurysms	47(47.5%)
Arterio-venous malformations/cavernous malformations	30(30.3%)
Cerebral bypass procedures	22(22.2%)
Location	
Infratentorial	62 (22.7%)
Supratentorial	211 (77.3%)
Race Category	
Non-White	59 (21.6%)
White	214 (78.4%)
Ethnicity Category	
Hispanic or Latino	26 (9.5%)
Not Hispanic or Latino	246 (90.1%)
Declined/Unknown	1 (0.4%)
Language Category	
English	249 (91.2%)
Non-English	24 (8.8%)
History of Chronic Pain	161 (59.0%)
History of a Depression/Anxiety Disorder	123 (45.1%)
History of Substance Abuse	60 (22.0%)
Preoperative Opioid Use	23 (8.4%)
Preoperative OME (*n* = 23)	55 [27; 64]
Length Of Surgical Procedure, Median [IQR]	331.6 [257; 399]
Hospital LOS, Median [IQR]	6.00 [4; 12]

Definitions: ASA: American Society of Anesthesiologists Classification; OME: Oral Morphine Equivalent; LOS: Length of Stay; IQR: Interquartile range.

**Table 2:** Factors Associated with Prescription of Opioids at the time of Hospital Discharge After Craniotomy for Tumor Resection/Vascular Repair.

	No Opiate Prescribed	Opiates Prescribed	Univariable Analysis *p*-Value	Multivariable Analysis OR [95% CI]	Multivariable Analysis *p*-Value
	*n* = 77	*n* = 196			
Age in years	54.3 (16.0)	51.3 (15.4)	0.158		
Sex			0.079		
Female	42 (54.5%)	131 (66.8%)			
Male	35 (45.5%)	65 (33.2%)			
ASA physical class			0.671		
1	2 (2.60%)	5 (2.55%)			
2	19 (24.7%)	57 (29.1%)			
3	44 (57.1%)	113 (57.7%)			
4	12 (15.6%)	21 (10.7%)			
Insurance carrier			0.569		
Commercial	38 (49.4%)	106 (54.1%)			
Non-commercial	39 (50.6%)	90 (45.9%)			
Type of surgical procedure			0.471		
Tumor	46 (59.7%)	128 (65.3%)			
Vascular	31 (40.3%)	68 (34.7%)			
Race			1		
Non-White	16 (20.8%)	42 (21.4%)			
White	61 (79.2%)	154 (78.6%)			
Ethnicity			0.871		
Declined/Unknown	0 (0.00%)	1 (0.51%)			
Hispanic or Latino	8 (10.4%)	18 (9.18%)			
Not Hispanic or Latino	69 (89.6%)	177 (90.3%)			
Language			0.771		
English	66 (85.7%)	163 (83.2%)			
Non-English	6 (7.79%)	15 (7.65%)			
History of chronic pain:			0.007		
0	42 (54.5%)	70 (35.7%)		Ref	
1	35 (45.5%)	126 (64.3%)		1.87 [1.06,3.29]	0.03
Psychiatry history:			0.625		
0	40 (51.9%)	110 (56.1%)			
1	37 (48.1%)	86 (43.9%)			
History of substance abuse:			1		
0	60 (77.9%)	153 (78.1%)			
1	17 (22.1%)	43 (21.9%)			
Preoperative opioid use	0.12 (0.32)	0.07 (0.26)	0.33		
0	68 (88.3%)	182 (92.9%)			
1	9 (11.7%)	14 (7.14%)			
Preoperative OME	55.0 (27.8)	75.2 (124)	0.637		
Length of Procedure	341 (115)	328 (102)	0.38		
Intraoperative ketamine			0.085		
infusion					
0	46 (59.7%)	140 (71.4%)			
1	31 (40.3%)	56 (28.6%)			
Maximum hospital pain score	7.25 (2.50)	7.61 (1.92)	0.197		
Total Hospital OME	495 (1287)	274 (348)	0.103		
Hospital LOS	15.6 (14.7)	8.07 (7.76)	<0.001	0.97 [0.93,1]	0.075
Hospital LOS category			<0.001		
1–4 days	8 (10.4%)	64 (32.7%)		Ref	
5–12 days	38 (49.4%)	105 (53.6%)		0.35 [0.15,0.8)	0.013
>12 days	31 (40.3%)	27 (13.8%)		0.12 [0.05,0.29]	<0.001

Abbreviations: OME: oral morphine equivalent; LOS: length of stay. For the calculation of the odds ratio: the “no opiates at discharge group” was considered as the reference group, and the “opiates at discharge group” was regarded as the outcome group.

## Data Availability

Not applicable.
